# Oxidative Stress and Poly(ADPribosyl)ation in Zebrafish Eyes After Exposure to Aluminium

**DOI:** 10.3390/biom15081169

**Published:** 2025-08-15

**Authors:** Anna Rita Bianchi, Valeria Guerretti, Alessandra La Pietra, Carmen Di Giovanni, Bruno Berman, Martina Falace, Teresa Capriello, Ida Ferrandino, Anna De Maio

**Affiliations:** 1 Department of Biology, University of Naples Federico II, Via Cinthia, 21, 80126 Naples, Italy; annarita.bianchi@unina.it (A.R.B.); valeria.guerretti@unina.it (V.G.); alessandra.lapietra@unina.it (A.L.P.); brunberm@libero.it (B.B.); marty.falace@live.it (M.F.); teresa.capriello@unina.it (T.C.); 2 Department of Pharmacy, University of Naples Federico II, Via Domenico Montesano, 49, 80131 Naples, Italy; cdigiova@unina.it

**Keywords:** aluminium toxicity, oxidative stress, PARP, PARG, zebrafish, DNA damage, antioxidant enzymes, eyes

## Abstract

Aluminium (Al) is a widespread environmental contaminant known to induce oxidative stress and genotoxic effects in aquatic organisms. While its neurotoxic properties are well documented, the molecular impact of Al on the visual system remains poorly understood. In this study, adult zebrafish (*Danio rerio*) were exposed to 11 mg/L Al for 10, 15, and 20 days to investigate the oxidative and genotoxic responses in ocular tissue. Activities of antioxidant enzymes superoxide dismutase (SOD) and catalase (CAT) were measured in eye supernatants to detect oxidative stress. Additionally, the activities of poly (ADP-ribose) polymerase (PARP) and poly (ADP-ribose) glycohydrolase (PARG) were assessed in tissue homogenates to evaluate oxidative DNA damage and repair processes. The results indicate that these enzymes respond to counteract the increased reactive oxygen species (ROS) induced by aluminium exposure. However, their activity may not sufficiently reduce ROS levels to fully prevent oxidative DNA damage, as evidenced by a significant rise in PARP activity during short exposure times. Over longer exposures, PARP activity returned to baseline, suggesting ocular cells may adapt to aluminium toxicity. We propose that this reduction in PARP activity is a cellular survival mechanism, as sustained activation can deplete energy reserves and trigger cell death. Finally, thin-layer chromatography confirmed that PARG facilitates the breakdown of poly (ADP-ribose) (PAR) into ADP-ribose, demonstrating the dynamic regulation of the PAR cycle, which is crucial to preventing parthanatos.

## 1. Introduction

Wild fish populations are subject to various environmental stressors, including climate change, pollution, and emerging pathogens [1,2,3,4,5]. Recent research has extensively investigated the molecular, physiological, and organismal effects of temperature variations and chemical pollutants on fish [6,7,8,9,10]. Much of this work has focused on identifying the responses to abiotic stresses and understanding how these responses integrate across different biological levels. This is essential for unravelling why certain species adapt and thrive under stress, while others may face extinction [2,11,12]. Adaptation to environmental stressors and survival depend on several factors, such as energy availability, food acquisition, metabolic efficiency, and the ability to maintain aerobic capacity. These factors facilitate the mobilisation of energy reserves required to offset the metabolic costs associated with defensive mechanisms [13,14]. The bioenergetic costs and stress responses—such as maintaining homeostasis, activating defence systems, and repairing cellular damage—are highly variable depending on the nature, duration, and intensity of the stressors [14]. Under short-term exposure to moderate stress, organisms can typically neutralise reactive oxygen species (ROS) through antioxidant defence systems and repair mechanisms [15,16,17,18], allowing some species to activate compensatory metabolic strategies that minimise oxidative damage [13].

To counteract ROS-induced damage and maintain cellular homeostasis, organisms have evolved a complex antioxidant defence network, which includes both enzymatic and non-enzymatic components [19]. Among the most effective enzymatic defences are superoxide dismutase (SOD) and catalase (CAT). SOD is the body’s primary detoxification enzyme and the most potent cellular antioxidant. An important endogenous antioxidant enzyme, it acts as a component of the first-line defence system against reactive oxygen species. It catalyses the dismutation of two superoxide anions (O_2_^−^) into hydrogen peroxide (H_2_O_2_) and molecular oxygen (O_2_), rendering the potentially harmful superoxide anion less dangerous in the process. CAT is a tetrameric protein with a molecular weight of 240 kDa, which is present in almost all living tissues that utilise oxygen. Using either iron or manganese as a cofactor, the enzyme catalyses the degradation or reduction of hydrogen peroxide (H_2_O_2_) to water and molecular oxygen. This completes the detoxification process that is mimicked by superoxide dismutase (SOD) [20]. Non-enzymatic defences comprise low-molecular-weight antioxidants such as glutathione (GSH) and thioredoxin, as well as dietary antioxidants, including vitamins C and E, carotenoids, and flavonoids [20,21,22]. However, prolonged exposure to multiple stressors, resulting in excessive oxidative stress, can cause irreversible cellular damage, including DNA damage, which has long-term consequences for organ function and species survival [23,24,25]. This ultimately impacts population persistence [26,27,28,29].

One of the initial responses to DNA damage is poly (ADPribosyl)ation, a reversible post-translational modification in which ADP-ribose units are added to target proteins, particularly those containing glutamate, aspartate, or lysine residues [30]. The ADP-ribose polymer (PAR), formed from sequential addition of ADP-ribose moieties from NAD^+^, can exceed 200 units, and exhibit multiple branching points [30]. Poly (ADP-ribose) glycohydrolase (PARG) catalyses the hydrolysis of PAR by cleaving ribose–ribose bonds, thereby releasing free poly (ADP-ribose) and ADP-ribose [31]. PARG has both endo- and exo-glycohydrolase activity, with a preference for the exo-glycosidic cleavage of the distal ADP-ribose units of the polymer [32,33]. In mammals, several PARG proteins, which are encoded by the same gene, are located in various cellular compartments alongside PARP proteins. Besides the very active nuclear PARG protein (110 kDa) and a short mitochondrial isoform (65 kDa), which are the predominant isoforms, several spliced variants have been described that lack exon 1 (102 kDa) or exons 1 and 2 (99 kDa) [34,35]. Complete deletion of PARG has been shown to cause embryonic lethality in *Drosophila* and to increase apoptosis in mice [36,37]. PARG also plays a key role in DNA repair [38], participates in the production of ATP from PAR, which is necessary for DNA replication [39], and it has been identified as a novel and critical component of single-strand break repair, acting alongside PARP1 [40]. PARG seems to be involved in regulation of NAD^+^ consumption during DNA repair [31]. Following DNA damage, PARP is known to use cellular NAD^+^ as a substrate, which is then rapidly recycled by PARG. Massive DNA damage causes PARP hyperactivation, resulting in the depletion of cellular NAD^+^ and ATP. This leads to the destabilisation of the mitochondrial membrane and the release of apoptosis-inducing factors (AIFs) into the nucleus [41,42]. AIF translocation culminates in parthanatos, an AIF-mediated form of apoptosis [43]. Increased parthanatos has also been reported in MNNG- or UV-treated PARG-deficient breast cancer cells in vitro [44]. This suggests that another function of PARG following DNA damage is to suppress cell death.

In zebrafish, two PARG isoforms (68 kDa and 87 kDa) were found in the brain, where they play a crucial role in preventing parthanatos cell death [31,45].

Poly (ADPribosyl)ation is catalysed by poly (ADP-ribose) polymerases (PARPs), a family of enzymes consisting of at least 18 members, which are encoded by different genes, and share homology in their conserved catalytic domain [46,47,48]. PARPs were found across various eukaryotic species [49,50].

Among them, the nuclear PARP1 and PARP2 are the most well-characterised enzymes, known for their ability to auto-modify themselves through PAR addition in response to DNA strand breaks [51]. While PARP1 and PARP2 are primarily involved in DNA repair, they also participate in numerous other cellular processes, including cell proliferation, transcription, chromatin remodelling, and apoptosis [52,53,54,55]. PARP1 and PARP2 activation is key in determining whether cells survive or die after DNA damage. In cases of mild DNA damage, PARP activation facilitates DNA repair, promoting cell survival. However, in response to severe DNA damage, the hyperactivation of PARP leads to NAD^+^ depletion and a subsequent loss of intracellular ATP reserves, driving cells towards necrosis or apoptosis [56]. Studies have also explored PARP modulation in response to abiotic stresses in plants [57,58] and marine organisms [59,60,61].

The activation of PARPs has also been linked to a range of human diseases, including cardiomyopathies, inflammatory disorders, cardiovascular diseases, diabetic complications, and neurodegenerative diseases [62], positioning PARPs as promising targets for anti-cancer therapies [63]. A detailed poly (ADPribosyl)ation system has been characterised in the zebrafish brain following aluminium exposure at 10, 15, and 20 days [45]. Aluminium is neurotoxic to fish, and its effects on zebrafish brain tissue resemble the pathology observed in humans with Alzheimer’s disease [64]. The highest PARP and PARG activities were recorded after 10 and 15 days of exposure, with a significant reduction in PARP activity after prolonged exposure. The initial increase in PARP activity was associated with oxidative DNA damage caused by aluminium, whereas the decrease in PARP activity at longer exposure times likely represents a cellular strategy to conserve energy and enable survival [45]. Pollutant toxicity has also been observed in the eyes of zebrafish, leading to visual dysfunction [65]. Fish eyes are particularly vulnerable to damage from pollutants because they are constantly exposed [66]. Visual impairments significantly increase the risks to individual survival, and can threaten the viability of entire populations, as fish become unable to escape predators or locate food [67,68]. Previous studies revealed histological retinal damage during initial aluminium exposure. Retinal degeneration occurred from 10 days of exposure, when increased PARP expression was also detected. Upregulation of genes involved in the regeneration process was observed at 15 days, and repair and recovery of retinal damage were observed at 20 days [69].

Based on these findings and following the protocol previously applied, this study used the same aluminium exposure durations to assess potential oxidative DNA damage in zebrafish eyes.

In detail, the present research aimed to evaluate the activities of superoxide dismutase (SOD) and catalase (CAT) in the eyes of adult zebrafish, both unexposed and exposed to 11 mg/L Al for 10, 15, and 20 days. This was carried out to investigate whether changes in antioxidant enzyme activities were linked to the onset of oxidative stress during exposure. Additionally, oxidative damage to genetic material was assessed indirectly by measuring PARP activity. PARG activity was also detected to assess whether the balance between poly (ADP-ribose) synthesis and degradation, essential for maintaining genomic integrity, was disrupted by exposure. Thin-layer chromatography (TLC) was employed to identify PAR digestion products and confirm the role of PARG in PAR turnover. Lastly, a molecular mechanism linking DNA repair and the maintenance of genomic integrity to antioxidant enzyme activity was proposed.

## 2. Materials and Methods

### 2.1. Zebrafish Breeding

Adult zebrafish were bred in glass tanks, at a constant temperature of 28 °C, a pH of 7.6, with a photoperiod 12:12 h light/dark; they were monitored daily in the Department of Biology, University of Naples Federico II. Fish were fed with commercial flakes (TetraMin Tropical Flake Fish^®^, Spectrum Brands, Blacksburg, VA, USA), commercial food supplemented with live *Artemia* sp. nauplii. The experimental protocols were conducted in accordance with the guidelines and policies dictated by European regulations on the wellness of animals employed for experimental purposes (Directive 2010/63/EU), and approved by the Italian Ministry of Health (licence 147/2019-PR).

### 2.2. Treatment and Sample Collection

The experimental conditions are the same as those already described in Capriello et al. [70]. Four groups of 11 fish each were set up in 25 L glass tanks. One group was exposed to water only (1/3 distilled water, 2/3 tap water) and used as control group (Ctrl), while the others were treated with 11 mg/L of Al for 10 (T10), 15 (T15), and 20 (T20) days, respectively. The water in the tanks was renewed daily, and food debris was removed. At the end of the treatments, the fish were euthanised with an overdose of ethyl 3-aminobenzoate methane sulfonate (MS-222 solution, Sigma-Aldrich, Munich, Germany, Cas No. 886–86–2) (300 mg/L), and their eyes were collected.

### 2.3. Sample Preparation

The eyes from the zebrafish that were unexposed (Ctrl) and exposed to Al were homogenised (Ultra Turrax T8, IKA-WERKE) in ice-cold homogenization buffer, containing 10 mM Tris-HCl (pH 7.5), 1 mM EDTA, 1 mM EGTA, 1 mM β-mercaptoethanol, 0.15 mM spermine, 0.75 mM spermidine, 1 mM PMSF, and 2 µg/mL protease inhibitor cocktail [69].

An aliquot of the homogenate was centrifuged at 10,000× *g* for 10 min at 4 °C. The supernatant was further centrifuged at 8000× *g* for 15 min to obtain a clear supernatant which was used to measure superoxide dismutase (SOD) and catalase (CAT) activities [71]. The Bradford assay (BioRad, Hercules, CA, USA) was used to measure the protein concentration.

### 2.4. Enzymatic Antioxidant Measurements

#### 2.4.1. SOD Activity

SOD catalyses the dismutation of the superoxide anion (O_2_^−^) into hydrogen peroxide and molecular oxygen. SOD activity was determined by a colorimetric assay method, which utilises a tetrazolium salt (WST-1) to detect superoxide radicals generated by the xanthine/xanthine oxidase system [72].

To perform the assay, 2 µg of the supernatant from the zebrafish eyes was added to a reaction mixture consisting of assay buffer (50 mM potassium phosphate, pH 7.4), xanthine oxidase (50 mM), and 1 mL WST-1 reagent, following the manufacturer’s instructions for the SOD assay kit (SOD kit CS1000, Sigma-Aldrich). The mixture was incubated for 20 min at 37 °C. SOD activity was measured based on the degree of inhibition of WST-1 formazan formation at 450 nm, using a microplate reader (Multiskan Sky, Thermo Scientific, Waltham, MA, USA). The SOD activity was expressed in U/mg of protein.

#### 2.4.2. CAT Activity

CAT enzyme activity was measured in the supernatant of zebrafish according to the manufacturer’s instructions of the commercial CAT colorimetric assay kit (CAT kit CAT100, Sigma-Aldrich). This method of analysis allows the measurement of hydrogen peroxide to remain after the action of catalase. Catalase converts hydrogen peroxide into water and oxygen. This enzymatic reaction is stopped with sodium azide, and the reaction mixture is subjected to a colorimetric assay. The colorimetric assay uses the substrate (3,5-dichloro-2-hydroxybenzenesulfonic acid), which binds oxidatively to 4-amino antipyrine in the presence of hydrogen peroxide and horseradish peroxidase (HRP) to give N-(4-antipyryl)-3-chloro-5-sulphonate-p-benzoquinone-monoimine, a quinone imine red dye which absorbs at 520 nm. In detail, 1 µg of the zebrafish eye supernatant was added to the reaction mixture, consisting of assay buffer (50 mM potassium phosphate, pH 7.0), 20 µL of hydrogen peroxide (10 mM). Subsequently, the reaction mixture was incubated for 15 min at room temperature. The reaction was stopped by adding stop solution. CAT activity was calculated based on the decrease in absorbance at 520 nm, resulting from the decomposition of hydrogen peroxide, using a microplate reader (Multiskan Sky, Thermo Scientific). The CAT activity was expressed in U/mg of protein.

### 2.5. Poly (ADP-Ribose) Polymerase (PARP) and Poly (ADP-Ribose) Glycohydrolase (PARG) Activity Assays

PARP activity assay was performed as described by Bianchi et al. [45]. Two aliquots of homogenate (20 μg of protein each) were incubated in the presence of 0.4 mM [^14^C]NAD^+^ (10,000 cpm/nmole) in a reaction mixture consisting of 0.5 M Tris-HCl (pH 8.0), 50 mM MgCl_2_, and 10 mM DTT. After 15 min incubation at 25 °C, the reaction was stopped by adding cold 20% trichloroacetic acid (TCA) (*w*/*v*). One of the aliquots was repeatedly washed with 7% TCA, and then filtered on Millipore filters (HAWPP0001, 0.45 μm).

The second aliquot was centrifuged at 7400× *g* for 5 min at 4 °C. After washing it in absolute ethanol, the resulting precipitate (consisting of both poly- and non-ADP-ribosylated proteins) was incubated for 15 min at 37 °C in PARG assay conditions, in a reaction mixture containing 100 mM Tris-HCl (pH 8.0), 10 mM DTT, and 20 µg of homogenate [59]. The reaction was stopped on ice, and the precipitates obtained by adding 20% TCA were washed with 7% TCA, and filtered on Millipore filters (HAWPP0001, 0.45 μm).

PARP and PARG activities were measured as the radioactivity of insoluble acid material using a liquid scintillation counter (LS 1701, Beckman, Milan, Italy) and were expressed as enzymatic milliunits per milligram of tissue (mU/mg).

### 2.6. Thin-Layer Chromatography (TLC) of Synthesised and Digested Poly (ADP-Ribose)

Two aliquots of homogenate (200 μg of protein each) from zebrafish eyes were incubated in a reaction mixture containing 0.5 M Tris-HCl (pH 7.5), 50 mM MgCl_2_, 10 mM DTT, and 0.5 mM [^14^C]NAD^+^ (100,000 cpm/nmol), for 15 min at 25 °C under PARP assay conditions [70]. The reaction was stopped on ice, and the proteins were precipitated by the addition of 30% TCA (*w*/*v*). Following centrifugation at 1200 × g at 4 °C for 20 min, the sample was washed three times with absolute ethanol. The resulting precipitate was then resuspended in a solution of 1 mM EDTA and 10 mM Tris-NaOH buffer at pH 12, and incubated for 3 h at 60 °C. Pure protein-free [^14^C]poly (ADP-ribose) was extracted three times with isoamyl alcohol/chloroform (1:24, *v*/*v*) [45].

An aliquot of extracted [^14^C]poly (ADP-ribose) (2000 cpm) was incubated in a reaction mixture containing 100 mM Tris-HCl (pH 8), 10 mM dithiothreitol and homogenate (200 µg) for 15 min at 37 °C in PARG assay conditions [45]. The reaction was then blocked at 4 °C, and the precipitable TCA fraction was washed with absolute ethanol.

Finally, the poly (ADP-ribose) and its degradation products by PARG were analysed by thin-layer chromatography (TLC) on PEI cellulose plates in 0.05 M ammonium bicarbonate [59]. Autoradiographic patterns of labelled poly (ADP-ribose) on TLC were acquired using a Phosphor-imager (model Fx, Quantity One Software https://www.bio-rad.com/zh-cn/product/quantity-one-1-d-analysis-software?ID=1de9eb3a-1eb5-4edb-82d2-68b91bf360fb (accessed on 23 July 2025), BioRad, Segrate, Italy).

### 2.7. Statistical Analysis

Statistically significant differences were assessed by Brown–Forsythe and Welch one-way analysis of variance (ANOVA) tests followed by Benjamini and Yekutieli multiple comparisons test using the GraphPad Prism 8 Software (Boston, MA, USA). The results were reported in the graph as the mean ± standard deviation (SD), and the minimum level of acceptable significance was *p* < 0.05.

## 3. Results

### 3.1. SOD and CAT Activities

**Figure 1:**
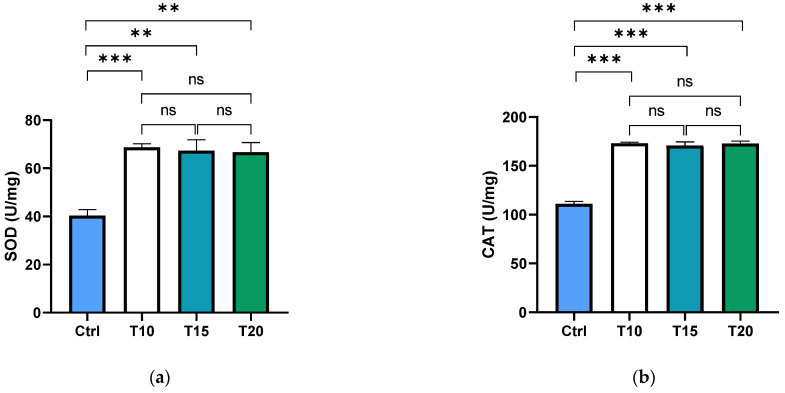
SOD (**a**) and CAT (**b**) activities were measured in the zebrafish eye supernatant of non-exposed (Ctrl), and aluminium-exposed fish for 10 (T10), 15 (T15), and 20 (T20) days. Data are presented as mean ± SD. Asterisks indicate statistically significant differences between the compared groups (** *p* ≤ 0.02; *** *p* < 0.001); ns indicates not significant.

Superoxide dismutase (SOD) and catalase (CAT) are key antioxidant enzymes that play a crucial role in shielding the ocular region from oxidative damage. As the primary defence against reactive oxygen species (ROS) in the eye, these enzymes help maintain redox homeostasis, thereby reducing the risk of oxidative stress-related conditions such as cataracts and macular degeneration [73,74]. In this study, the activity levels of SOD and CAT were assessed in the supernatants of dissected zebrafish eyes following aluminium exposure for 10, 15, or 20 days. Both SOD (Figure 1a) and CAT (Figure 1b) activities were significantly elevated in all aluminium-exposed groups compared to the control. However, no significant differences were observed among the different exposure durations.

### 3.2. PARP and PARG Activities

**Figure 2:**
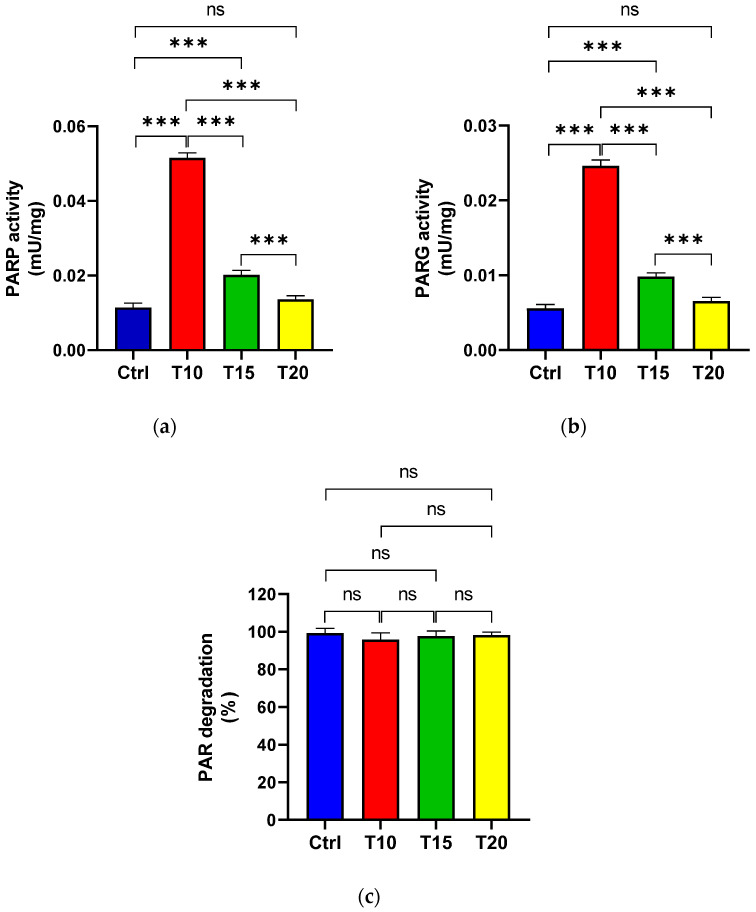
PARP (**a**) and PARG (**b**) activities, as well as the percentage of PAR degradation (**c**), were measured in zebrafish eyes from control (Ctrl), and aluminium-exposed groups at 10 (T10), 15 (T15), and 20 (T20) days. Data are presented as mean ± SD. Asterisks indicate statistically significant differences between the compared groups (*** *p* < 0.001); ns indicates not significant.

PARP (Figure 2a) and PARG (Figure 2b) activities were assessed in eye homogenates from zebrafish either unexposed (Ctrl) or exposed to aluminium for 10, 15, and 20 days, in order to evaluate PAR synthesis and its degradation. Additionally, the percentage of PAR degradation was measured (Figure 2c). The highest PARP activity was observed at T10 (Figure 2a). Although enzyme activity decreased at T15 compared to T10, it remained significantly higher than the control level. By T20, PARP activity levels had returned to baseline and were comparable to those of the control group. PARG activity followed a similar pattern to PARP activity (Figure 2b). Across all eye homogenate samples, PAR degradation exceeded 95% (Figure 2c).

### 3.3. TLC of Poly (ADP-Ribose) and Its Degradation Product

**Figure 3:**
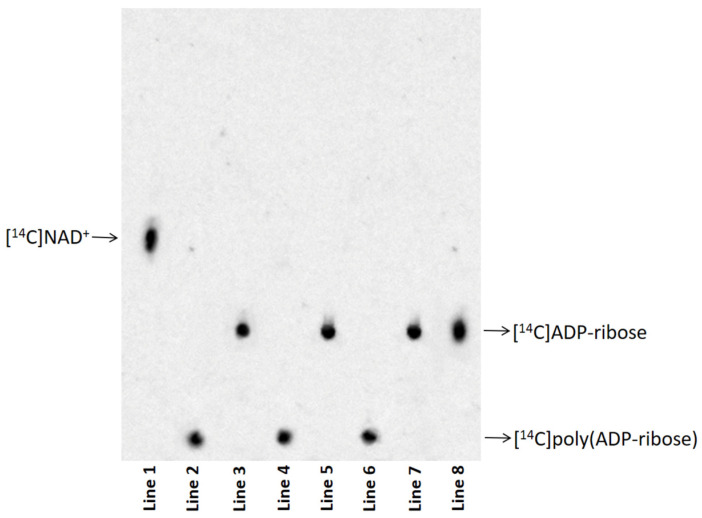
Protein-free [^14^C]poly (ADP-ribose) (400 cpm) purified from eye homogenates exposed to Al for 10 (line 2), 15 (line 4), and 20 (line 6) days and its degradation product by PARG (line 3, line 5, and line 7). Nucleotide standards were: [^14^C]NAD^+^ (400 cpm) (line 1) and [^14^C]ADP-ribose (400 cpm) (line 8). The original image is shown in Supplementary Material Figure S1.

[^14^C]poly (ADP-ribose) purified from eye homogenates exposed to Al for 10 (line 2), 15 (line 4), and 20 (line 6) days and their respective degradation products by PARG (line 3, line 5, and line 7) were analysed via thin-layer chromatography. [^14^C]NAD^+^ (line 1) and [^14^C]ADP-ribose (line 8) were used as nucleotide standards (Figure 3). The disappearance of the radioactive signal at the TLC loading point, and the simultaneous appearance of a signal co-migrating with the standard ADP-ribose were demonstrated in all analysed samples (Figure 3). These results indicated that the protein-free polymers were completely degraded to ADP-ribose.

## 4. Discussion

Aluminium (Al) is one of the most abundant metals in nature. It represents a significant environmental threat due to its ability to exert toxic effects on both terrestrial and aquatic organisms [75]. Many studies in fish have demonstrated that aluminium exposure can disrupt physiological functions [76], interfere with biochemical pathways [77], impair fertility [78], inhibit growth [79], and increase mortality [80].

In zebrafish embryos, aluminium exposure alters redox homeostasis and induces apoptosis [81]. In adult zebrafish, aluminium causes histopathological alterations in muscle [82], gill tissues [83], and induces behavioural and biochemical changes in the brain [84]. This metal increases, in fact, anxiety in fishes exposed for 15 days, and causes a time-dependent increase in ROS, while oxidative damage varies differently with an increase appreciable just after few days of treatment [84]. Aluminium is also considered a contributing factor to neurodegenerative diseases. Previous research has shown that aluminium exposure leads to neurodegenerative processes and alters the expression of marker genes associated with Parkinsonism in the brains of zebrafish [70].

Recently, we characterised the poly (ADPribosyl)ation system in adult zebrafish brains, in which the highest PARP and PARG activities were measured at 10 and 15 days of exposure to 11 mg/L of Al [45]. In this study, we have assumed that PARP activation is related to oxidative DNA damage caused by this metal, while PARG activation avoids PAR accumulation, which is known to inhibit PARP [45,85]. Conversely, PARP activity reduction, detected at longer exposure times, was considered a strategy adopted by neuronal cells to conserve energy and to assure survival [45].

Oxidative DNA damage has also been demonstrated in zebrafish eyes, where increased expression of PARP1 and PARP2 was observed at only 10 days of aluminium exposure [69]. Given that oxidative stress, primarily through increased ROS, is a major driver of oxidative DNA damage, the present study aimed to assess the activities of two key antioxidant enzymes, SOD and CAT, in supernatants of zebrafish eyes following exposure to 11 mg/L Al for 10, 15, and 20 days. In parallel, PARP activity was measured in tissue homogenates to evaluate the DNA damage response. The marked increase in SOD, CAT, and PARP activities at 10 days of exposure supports the hypothesis that oxidative stress induced by aluminium causes genotoxic damage, triggering PARP activation. During this period, antioxidant enzymes attempt to mitigate ROS elevation but may be insufficient to fully prevent genotoxic stress, as reflected by sustained PARP activation. The activation of poly (ADPribosyl)ation in the zebrafish eyes reinforces PARP’s function as a DNA damage sensor involved in repair and genomic stability maintenance [45,68,69].

The present data appear to be consistent with those observed in a study on the brain of zebrafish treated with 11 mg/L of Al for 20 days. In the brain of this fish, the activation of poly (ADPribosyl)ation and the subsequent increase in PAR synthesis provided indirect evidence of genotoxic damage caused by brief exposure [45]. Furthermore, it has already been demonstrated that short exposure times induced oxidative damage in zebrafish gill tissue, where increased levels of lipid and protein oxidation were observed [83]. A significant increase in the activity of certain antioxidant enzymes was also detected in zebrafish muscle tissue [82].

At extended exposure durations (20 days), the observed reduction in PARP activity to control levels suggests ocular adaptation to aluminium toxicity, likely through energy conservation mechanisms, that prevent depletion of NAD^+^ and ATP pools [45].

Since PARP is a major cellular energy consumer, it is reasonable to assume that downregulation of its activity ensures maintenance of the energy homeostasis, essential for cell survival [45,58].

The evidence that the highest PARP activity occurs at 10 days of exposure, while retinal degeneration phenomena become evident at 15 days [69], confirms that genotoxic stress precedes histological damage in the eye. The decrease in PARP activity, together with retinal regeneration at 20 days of exposure, instead, is indicative of restored histological and molecular damage [69].

Our previous studies have also shown that oxidative damage is significantly reduced in the gills and brain of zebrafish exposed to aluminium at prolonged exposure times (20 days) [70,83,84]. During these periods, the histological alterations, observed at short exposure times, return close to normal. This stabilisation/improvement of conditions has also been observed in zebrafish exposed to other pollutants [86,87,88]. It has been linked to fish’s ability to adapt to stressful environments by activating efficient detoxification mechanisms, enabling them to survive [89].

Therefore, PARG activity, responsible for hydrolysing PAR into ADP-ribose, was also measured in zebrafish eyes. The highest level of PARG activity was detected at 10 days of exposure, when PARP activity was also at its maximum. These results suggest that PARG activity plays an important role in the proper functioning of the poly (ADPribosyl)ation system, which requires a balance between PAR synthesis and its degradation [45]. This balance is crucial to avoid PAR overaccumulation, which inhibits PARP activity, compromises DNA repair, and thereby promotes cell death through parthanatos, a form of AIF-mediated, caspase-independent apoptosis [90]. We therefore hypothesise that PARG plays a key role in regulating the consumption of NAD^+^ and suppressing cell death in the eyes of zebrafish exposed to aluminium [31].

## 5. Conclusions

For the first time, we report the presence of both poly (ADP-ribose) polymerase (PARP) activity, responsible for the synthesis of poly (ADP-ribose), and poly (ADP-ribose) glycohydrolase (PARG) activity, which mediates its degradation, in homogenates of zebrafish eyes following exposure to aluminium (11 mg/L) for 10, 15, or 20 days. The concurrent activation of the poly (ADPribosyl)ation pathway, and elevated antioxidant enzyme activities observed at 10 days, strongly suggests the occurrence of genomic damage resulting from oxidative stress. At later time points, we hypothesise that the resulting decrease in PARP activity is related to the maintenance of energy homeostasis, which allows cells to adapt to living exposure to aluminium.

Finally, the balance between PAR synthesis and degradation at every exposure time, would avoid PARP inhibition due to PAR accumulation and parthanatos induction.

## Data Availability

The results presented in this study are included in the article. Further inquiries can be directed to the corresponding author.

## Supplementary Materials

The following supporting information can be downloaded at: https://www.mdpi.com/article/10.3390/biom15081169/s1, Figure S1: The original picture of Figure 3.
