# Supplementary figures and images for: Oxidative Stress and Poly(ADPribosyl)ation in Zebrafish Eyes After Exposure to Aluminium
Source: Biomolecules. 2025 Aug 15;15(8):1169. doi: 10.3390/biom15081169 (PMC12383955; doi:10.3390/biom15081169)

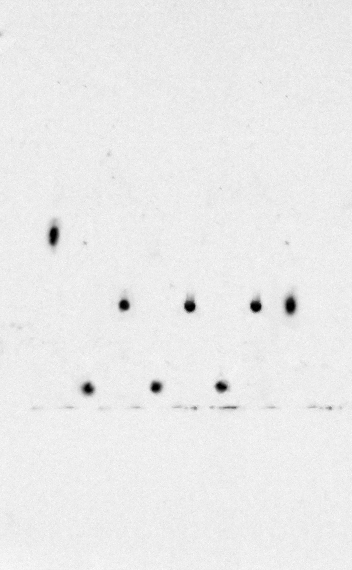

Supplement: Supplementary file 1 [file biomolecules-15-01169-s001.zip › Figure S1.tif]
